# Sleep-related metrics that predict training intensity: an exploratory study in women's college athletics

**DOI:** 10.3389/fspor.2026.1849511

**Published:** 2026-09-17

**Authors:** Amelia E. Frey, Carter Stoke, Cat Provenzano, Jason Fanning

**Affiliations:** Department of Health and Exercise Science, Wake Forest University, Winston-Salem, NC, United States

**Keywords:** athletics, exertion, RPE, sleep, WHOOP

## Abstract

**Background:**

In recent years, the development of wearable monitoring technologies has expanded objective assessments of workload and fatigue, predominantly via heart rate (HR)-based markers (e.g., heart rate variability, resting heart rate). There are also perception-based markers of exertion commonly used in high-performance training settings, such as rating of perceived exertion (RPE) scales. Previous research has shown that these markers do not measure identical but rather complementary aspects of exertion, but limited data exist on the relationships between objective and subjective markers at the daily level; data which may be especially important for understanding how to best monitor for effort or overtraining. This study will characterize both HR- and perception-based markers of exertion at the daily level in women's college athletes. Additionally, given the powerful influence of sleep on both athletic performance and perceptions of physical exertion, we further explored how sleep behaviors at the within- and between-person level relate to daily assessments of subjective and objective exertion.

**Methods:**

Thirteen women's college athletes continuously wore a WHOOP activity monitor for this two-week observational study. RPE was self-reported within 30 min of completing a training session or competition and prior to viewing WHOOP data.

**Results:**

A series of descriptive statistics and Pearson correlation were conducted to describe the relationship between RPE and activity strain on a daily level among the population studied. A series of linear mixed-effects models demonstrated worse sleep on average (i.e., more debt, poorer sleep performance) was associated with higher HR-based markers of exertion; however, within-person elevations in sleep consistency, but more sleep need on average was associated with higher HR-based measures of exertion and lower perceptions of exertion.

**Conclusions:**

Findings support the need for additional research to better understand the drivers of daily markers of exertion to enhance the health and performance of athletes.

## 1 Introduction

Understanding the factors that contribute to optimal performance is imperative for athletes, as well as for those who train or coach athletes. Maximizing athletic performance requires a balance between repeated exertion during training sessions (i.e., training volume) and appropriate recovery (1–3) Exertion refers to energy expenditure during physical activity and is measured using physiological markers such as oxygen consumption, heat production, or, commonly, metrics derived from heart rate (HR) such as time spent at varying intensities (4, 5). Exertion can also be measured from a psychological perspective. For instance one's rating of perceived exertion (RPE) captures one's subjective experience of exertion and recovery (6).

Repetitive high-exertion training sessions contribute to muscle breakdown and can place athletes into a period of functional overreaching, which is necessary for endurance development and muscle growth (7). However, periods of functional overreaching are only effective when balanced with adequate recovery. Repetitive high exertion without proper recovery can contribute to overtraining, illness, injury, and overall decreased athletic performance and quality of life (8–10) Thus, research has sought effective ways of capturing exertion, with both objective and subjective methods gaining prominence (4, 11). Understanding the degree to which HR-based and perception-based markers of exertion explain similar or different aspects of training load and fatigue status can assist those who seek high levels of fitness and performance outcomes to modify their training and recovery behavior (5).

Research on objective markers of training and recovery has benefitted by widespread uptake of consumer wearable fitness devices (WFDs), with widely-used products including those produced by companies like WHOOP, Fitbit, Garmin, and Apple (12–14). WFDs are now frequently used by Division 1 and professional athletes in high-performance settings; the primary function of WFDs in these settings is to provide data to both athletes and coaches, which enables behavior modification with regard to training and recovery (15). The ease of data collection of HR-based metrics from WFDs allows athletes and coaches to capture the physiological facets of individual exertion and recovery to better personalize training and optimize performance (16, 17).

It has long been recognized that objective markers of training and recovery do not capture the full picture, and that it is necessary to consider athletes’ perception-based markers as subjective experiences of exertion and recovery in conjunction with objective measures (1, 2). Rating of perceived exertion (RPE) is a perception-based marker typically presented as a 1–10 scale in which an individual self-assesses their level of exertion, or how hard they feel to be working with respect to their abilities. Indeed, one's perceptions of their own exertion and recovery capture both shared (e.g., increases in heart rate and energy expenditure) and unique (e.g., perceptions of heightened pain due to the influence of stress) information relative to objective markers. The measurement of fatigue offers a useful analogy to the measurement of exertion. Thorpe and colleagues reviewed methods for monitoring fatigue status in elite athletes. The authors noted that fatigue is multifactorial in nature, arising from training load, psychological stress, and injury. Thus, no single metric is likely to sufficiently explain an athlete's fatigue status. Instead, they emphasize the need for a combination of objective and subjective markers of fatigue (17). A body of evidence supports the recommendations by Thorpe and colleagues to utilize subjective measures to measure training and recovery status, given the host of influences on these outcomes. For instance, subjective experiences that interact with training and recovery are affected by psychological characteristics such as stress-management/coping skills and level of mindfulness as well as one's affective valence (9). Personality factors such as extraversion (marked by a tendency toward interactions with others) have been found to relate negatively to RPE, such that individuals with higher levels of extraversion and stimulation-seeking behaviors tend to perceive physical activity as relatively less exertional, especially at lower levels of intensity. Conversely, variables such as depression, anxiety, and neuroticism often manifest as higher levels of trait anxiety and a more exaggerated appraisal of challenging stimuli such as physical exertion (especially at higher intensities) (18).

One important but understudied consideration in the measurement of subjective influences on training and recovery is that many of the most widely-cited variables (e.g., stress, affect, training load) are highly dynamic. For instance, for student athletes, acute academic stressors or social pressures (e.g., to socialize rather than sleep) may drive acute discrepancies between objective and subjective markers of exertion. An advantage of widespread use of WFDs is access to temporally-dense data capturing both training intensity and recovery, which may offer insights into both general trends (i.e., between-person differences), as well as how daily fluctuations in objective markers of exertion may relate to daily fluctuations in perceptions of exertion. Moreover, contemporary WFDs used widely in athletics (e.g., those produced by WHOOP) provide data on sleep—a powerful driver of daily stress and recovery—allowing for investigation on how general sleep behaviors, or daily changes in sleep behavior, may affect objective and subjective markers of training and recovery. Sleep is an essential time in which the body adapts to and recovers from physiological exertion, repairing micro-tears in muscle and facilitating removal of metabolic waste, for example. Thus, we would expect sleep behaviors to demonstrate correlation to subjective and objective measures of exertion, which are both dependent on the recovery processes that occur during sleep (19). Due to the role of sleep in facilitating recovery, sleep metrics were included to explore the extent to which sleep behavior was associated with objective and subjective measures of exertion. Such information may be highly valuable to athletes and coaches, potentially better elucidating the impact of both chronic and acute behavioral choices on training and performance. At present, we are unable to identify any studies that have (1) characterized daily-level WFD-derived markers of training load alongside daily self-reported exertion during training in college athletics, and (2) investigated the relationships between nightly sleep behaviors and objective and subjective markers of exertion. Therefore, the purpose of this exploratory pilot study was to characterize both HR- and perception-based markers of exertion at a daily level for women's college athletes, and to examine within-person relationships between these constructs. Additionally, we explored the relationship between subjective and objective markers of exertion and sleep to explore the extent to which sleep provides similar or unique prediction of daily perceived and objective exertion.

## 2 Methods

### 2.1 Participants

Participants were women's college athletes from a university in the southeastern United States and were eligible if they were an active member of the soccer or field hockey team (all rostered members were considered active members regardless of playing time or red-shirt status) and were actively using the team-provisioned WFD (i.e., the WHOOP version 4.0). Eligible participants were free of serious injury, able to compete in team training sessions and competitions, and able to continuously wear their WHOOP for a two-week data collection period. This study was approved by the Institutional Review Board (IRB # IRB00025628) and all participants signed an informed consent prior to engaging study procedures.

### 2.2 Study design

This was a two-week observational study. Data collection occurred during the month of September 2024. Participants were instructed to wear their WHOOP continuously for a two-week period and to submit a rating of perceived exertion (RPE) evaluation via a mobile app within 30 min of completion of a training session, workout, or competition as per protocol for both teams. Participants were instructed to submit their RPE prior to reviewing their WHOOP data.

### 2.3 Measures

#### 2.3.1 Rating of perceived exertion

The Borg rating of perceived exertion (RPE) scale captures the subjective experience of exertion during physical activity. Scores originally range from 6 (*no exertion*) to 20 (*maximal exertion*) to approximate 10% of a healthy young adult's heart rate at a given intensity. The RPE scale was later modified to range from 0 to 10 for ease of understanding (7). In this study, RPE is specific to each training session and documented as a whole-number value on the adjusted Borg scale. The scale anchors were standardized and explained to participants using layman's terms such that a rating of 0 equates to “not present at session” and to 10 represents one's “hardest session ever*”*.

#### 2.3.2 HR-based markers of exertion

All participants utilized WHOOP (version 4.0) during the assessment period. The WHOOP Performance Optimization System (WHOOP Inc.) is a wrist-worn multi-sensor (tri-axial accelerometer, green light LED-derived photoplethysmography (PPG), capacitive touch sensor, and ambient temperature sensor) device (20). The primary marker of exertion utilized herein was daily strain. Strain is a proprietary measure of an individual's exertion during a 24-hour period accumulated from training sessions and throughout the day, and is derived as an aggregate of cardiovascular, muscular, and cognitive load, scaled from 0 to 21 (21). Training sessions varied in type and frequency dependent upon the sport and included both practices and work with the team's respective strength and conditioning trainer. Activity strain is a metric that more specifically describes the cardiovascular load of a given exercise bout (such as a training session or game) and was analyzed alongside RPE in this study.

#### 2.3.3 Sleep Metrics

WHOOP 4.0 uses algorithms that incorporate data from accelerometers, PPG, and skin temperature sensors to detect sleep onset times and classify sleep stages [awake, light, deep slow wave or rapid eye movement]. Durations of each stage were included as variables. Additionally, WHOOP tracks the following sleep metrics that were included in the analysis: sleep performance, sleep need, sleep debt, sleep consistency, awake duration, asleep duration, and respiratory rate. Sleep performance is reflected as a percentage with total sleep duration as the numerator and sleep need as the denominator. Sleep need is a recommended duration of sleep that varies night-to-night and is calculated based on factors such as baseline sleep (estimated using age and gender) daily strain, recent sleep debt, and naps. Sleep debt reflects the deficit (in minutes or hours) between the duration of one's sleep need compared to the duration of sleep achieved. Sleep consistency captures the extent to which an individual maintains a similar, consistent pattern of sleep and wake times throughout the week. Respiratory rate is a measure of the average number of respirations per minute over the course of a sleep cycle (22). These metrics are incorporated into the algorithm that WHOOP uses to present overall sleep performance scores (expressed as a percentage) to the user.

### 2.4 Statistical analysis

The primary purpose of these analyses was to describe RPE and activity strain measured at the daily level among a sample of college athletes. To that end, we first computed a series of descriptive statistics [i.e., mean (standard deviation) for continuous variables, *n* (%) for count variables] capturing sample characteristics, and measures of central tendency and distribution both between subjects (i.e., based upon an individual's average for each variable) and within subjects (i.e., statistics were computed within the individual and then summarized). Within-subject range for each marker is also depicted on a waterfall plot to illustrate the individual variability in each measure. A Pearson correlation was conducted to investigate the overall correlation between RPE and activity strain values without consideration for the clustered nature of the data. Next, to estimate the proportion of variability explained by differences within and between subjects via intraclass correlation (ICC), we fit a series of null linear mixed effects models, including RPE or activity strain as the dependent variable, and including a random intercept. A mixed effect model was then fit predicting activity strain by RPE to capture the strength of the relationship between these variables while accounting for the nested nature of the data. Finally, to illustrate the extent to which these variables capture similar or unique facets of athletic exertion, we fit series of linear mixed effects models to investigate whether various sleep-related WHOOP metrics (i.e., sleep debt, performance, consistency) were associated with markers of intensity during training or competition (i.e., energy expenditure, activity strain, RPE). Specifically, each model investigated the relationship between daily markers of exertion and sleep from the prior night. For each predictor of interest, we included both the individual's mean score and each session's difference from the mean, allowing for investigation of general between-person effects and within-person differences respectively. In each mixed effect model, effects were estimated using maximum likelihood. Note that as a small and exploratory study, we prioritized type-II error and as such did not adjust for multiple comparisons. Histograms of residuals were inspected to verify normality, and scatter plots of predicted by residual values were utilized to confirm homoscedasticity. Statistical significance was established at *p* < 0.05. All analyses were conducted in SPSS Version 29 (171).

## 3 Results

### 3.1 Participant characteristics

Fifteen women's college student-athletes were recruited to participate in the study. Two participants dropped out prior to study completion due to injury and voluntary termination of WHOOP use, respectively, and an additional two participants did not have overlap between self-reported exertion and WHOOP data. As a result, 11 participants were included in analyses. In total, there were 147 days with activity-related observations and 102 days with sleep-related observations. On average, participants provided 13.36 ± 5.41 activity-related and 9.27 ± 3.72 sleep-related observations. Participants were 20.69 ± 1.31 years of age on average, and the majority of participants were White (72.7%). Participant characteristics are displayed in Table 1.

**Table 1:** Participant characteristics.

Participant characteristics	*N* = 11
Age, y: M (SD)	20.69 (1.31)
Weight, lbs: (SD)	137.18 (11.97)
Height, in: (SD)	66.00 (2.76)
Race: *N* (%)	
White	8 (72.7)
Black or African American	1 (9.1)
Asian	1 (9.1)
Other	1 (9.1)
Sport: *N* (%)	
Field hockey	5 (45.5)
Women's soccer	6 (55.5)

M, mean; SD, standard deviation; y, years; in, inches; lbs, pounds.

Waterfall plots for RPE and activity strain are depicted in Supplementary Figure 1. All observations across RPE and strain variables are depicted in Supplementary Figure 2. The mean of each individual's average RPE score was 4.37 (*SD* = 1.14; *range=*2.17–6.29). The mean of each person's average activity strain was 10.25 (*SD* = 1.20; *range=*9.10–12.53). Across all observations, RPE and activity strain were weakly, but positively correlated (*r* = 0.29; see Supplementary Figure 2).

The ICC for strain was 0.03, indicating that 97% of the variance in strain values was within-subject. The ICC for RPE was 0.17, indicating that 83% of the variance in RPE was within-subject. Additionally, RPE was a significant predictor of strain (B = .535, *p* < .001), accounting for 12.1% of the variance in activity strain.

### 3.2 Relationships between sleep and RPE or strain

Regarding objective markers of exertion, models revealed that a person's average sleep debt was positively associated with activity strain (B = 0.03, *p* = 0.04), such that each one-point increase in average sleep debt was associated with a 0.03-point increase in activity strain. A person's average sleep performance was negatively associated with activity strain (B = −0.71, *p* = 0.05), such that each one-point increase in average sleep performance was associated with a 0.71-point decrease in activity strain. Finally, average sleep need was negatively associated with RPE (B = -.02, *p* = 0.05), such that each one-point increase in average sleep need was associated with a 0.02-point decrease in RPE. Sleep consistency was not associated with either activity strain or RPE. No other sleep variables were associated with RPE or activity strain at the within-person level. Taken together, these results suggest that worse sleep on average (i.e., more debt, poorer performance, more sleep need) was associated with higher HR-based markers of exertion (activity strain), but lower perception-based markers of exertion (RPE). Additionally, sleep consistency at the within-person level (i.e., the difference between each observation and the person mean) was negatively associated with RPE (B = -.09, *p* = 0.017) such that a one-point increase in consistency relative to one's average is associated with 0.09-point lower rating of perceived exertion. Results of linear mixed effects are depicted in Supplementary Tables 1–S5.

## 4 Discussion

The primary objective of this study was to characterize relationships between HR- and perception-based markers of exertion at the daily level for women's college athletes. In accordance with findings from previous studies (11, 23) we observed a significant and positive correlation between HR- and perception-based markers of exertion overall, such that individuals perceived their training or performance exertion higher on days when they experienced more strain. The study by Wallace and colleagues found a significant and positive correlation between session-RPE and HR-based methods (r = 0.55–0.94; *p* < 0.05) in well-trained swimmers. Our observed correlations were not as strong, which may be explained by contrasting devices (WHOOP and HR-monitor only), different populations, and the nature of the sport (for example, external factors such as temperature could impact both HR and RPE in soccer and field hockey sessions as compared to perhaps a lesser effect in swimming sessions). One would expect this correlation to be low given the multitude of factors that contribute to both HR- and perception-based exertion, and the fact that perception of exertion in athletic training settings is perhaps more nuanced than perception of exertion in experimental settings (i.e., exercise tests performed on a treadmill). The correlation observed between HR- and perception-based measures of exertion corroborates previously published findings. For instance, Scherr and colleagues reported rating of perceived exertion to be strongly correlated with heart rate (r = 0.74, *p* < 0.001) and blood lactate (r = 0.83, *p* < 0.001) in a cohort study of 2,560 Caucasian men and women (11). Interestingly, we also observed a considerable amount of within-subject variability in both HR- and perception-based markers of exertion. Moreover, RPE accounted for only 12% of the variance in activity strain, supporting that these constructs were variable on the day-to-day level and captured both shared and unique information within this exploratory sample (17). Taken together, these findings are unsurprising but support the importance of adopting a multifactorial and daily approach to assessing exertion in athletes.

Our findings related to sleep offer an initial glimpse into the value of daily data for better understanding the relationship between behaviors like sleep and objective and subjective markers of exertion. The overall level of sleep quality of the sample was average in comparison to the general population of the target demographic studied. Descriptive statistics of sleep need reflect consistently high exertion of the sample overall, as higher strain increases sleep need even in the absence of accumulated sleep debt. In general, we found that an individual's average sleep debt and performance were associated with objective markers of exertion, such that those who tended to exhibit worse sleep also tended to have higher strain. Findings were more mixed for RPE whereby a greater sleep need on average was associated with lower perceived exertion. Conversely, days with higher consistency were associated with lower subsequent RPE; the only within-person effect that reached statistical significance. The finding that sleep was associated with higher strain but lower perceived exertion is an interesting one that warrants further investigation. If such a finding holds in future investigation—that those who sleep poorly may exert more but perceive less—it may suggest an increased risk of overexertion and warrant the application of behavioral intervention to improve sleep. Fortunately, evidence suggests that athletes may be responsive to sleep coaching. Driller and colleagues found that individualized sleep education significantly increased both subjective and objective measures of sleep outcomes in elite male cricket athletes– specifically sleep efficiency, sleep onset variance, and sleep onset latency (24).

Taken together, these early findings suggest there is value for additional research motivated by a desire to understand the factors that affect both objective and subjective markers of exertion. For instance, given the degree of within-person variability in both RPE and strain we observed, future research utilizing methods such as ecological momentary assessment to identify factors driving momentary scores is warranted. Ecological momentary assessment typically involves the real-time assessment of self-reported phenomena multiple times during the day, often via smartphone (25). Modeling the relationships between objectively-assessed sleep, daily strain and perceived exertion, and momentary ratings of constructs such as fatigue or stress may help to guide future coaching practices designed to optimize training and performance.

### 4.1 Strengths and limitations

One strength of this study is that it is among the first to examine relationships between RPE and objective markers of exertion and sleep at the daily level among collegiate athletes. It also leveraged a WFD that provides relatively high-quality sleep (AIBS) and heart-rate derived metrics (13). There are also several limitations to this study. Most importantly, as an initial exploration of daily perceptions of exertion, no *a priori* power analysis was conducted. Moreover, we recruited a small convenience sample that lacked sport, ethnic, and gender diversity. As such, future work with larger and more diverse samples is warranted to establish the reliability of the findings presented herein. A larger, more generalizable sample would be beneficial to investigate the difference in HR- and perception-based exertion, and the relationship of each marker type, respectively, with sleep-related metrics for more varied athletic and sociodemographic groups. The nature of WHOOP-derived metrics as proprietary and not fully described in terms of their underlying algorithms also serves to limit reproducibility and interpretability of this exploratory study. Finally, the WHOOP brings a relatively high cost, given its nature as a subscription-based service, which may limit the utility of WHOOP-based findings to inform real-world programming.

## 5 Conclusion

The results observed in this study indicate that objective and subjective markers of exertion, recorded at the daily level, varied substantially within the individual and capture both shared and unique information related to exertion. We also observed that worse overall sleep (more debt, poorer performance, more sleep need) is associated with higher HR-based and lower perception-based markers of exertion. Moreover, within-person differences in sleep inconsistency was associated lower perception-based markers of exertion; findings that point to the need for additional research on whether those with poor acute or chronic sleep are more prone to overtraining. These findings support the value of additional research to better understand the drivers of daily markers of exertion to enhance the health and performance of athletes.

## Data Availability

The raw data supporting the conclusions of this article will be made available by the authors, without undue reservation.
