# Supplementary material for: Sleep-related metrics that predict training intensity: an exploratory study in women's college athletics
Source: Front Sports Act Living. 2026 Sep 17;8:1849511. doi: 10.3389/fspor.2026.1849511 (PMC13627433; doi:10.3389/fspor.2026.1849511)
Supplement: Supplementary file 1 [file Datasheet1.pdf]

Supplementary Material

1 Tables

Supplementary Table 1. *Participant Characteristics*

**Participant Characteristics**	**N=13**
Age, y: M (SD)	20.30 (1.18)
Weight, lbs: (SD)	137.62 (11.17)
Height, in: (SD)	66.38 (2.69)
Race: N (%)	
White	9 (69.2)
Black or African American	1 (7.7)
Asian	2 (15.4)
Other	1 (7.7)
Sport: N (%)	
Field Hockey	5 (38.5)
Women's Soccer	8 (61.5)

Note: M=Mean, SD= Standard deviation y= years, in=inches, lbs=pounds

Supplementary Table 2. *Linear Mixed Effects*

Measure of Intensity	Predictor	General (B)	Acute (B)
Activity Strain	Sleep Debt	0.03*	0.00
Activity Strain	Sleep Performance	-0.71*	0.10
RPE	Sleep Consistency	-0.03	-0.09*
RPE	Sleep Need	-0.02*	-0.01

Note: * indicates results are significant (p<.05)

RPE is defined as the athlete's rating of exertion on a 0–10 scale for any given session.

2 Figures

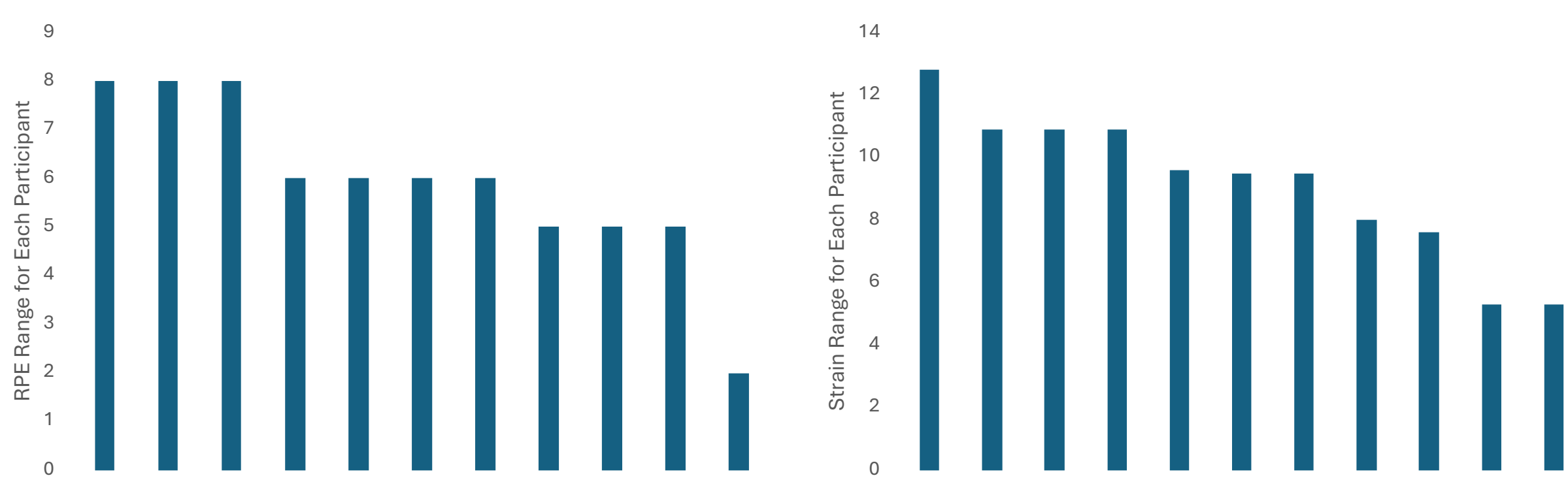

Supplementary Figure 1. Waterfall plots depicting individual participant ranges for rating of perceived exertion (RPE; left) and activity strain (right).

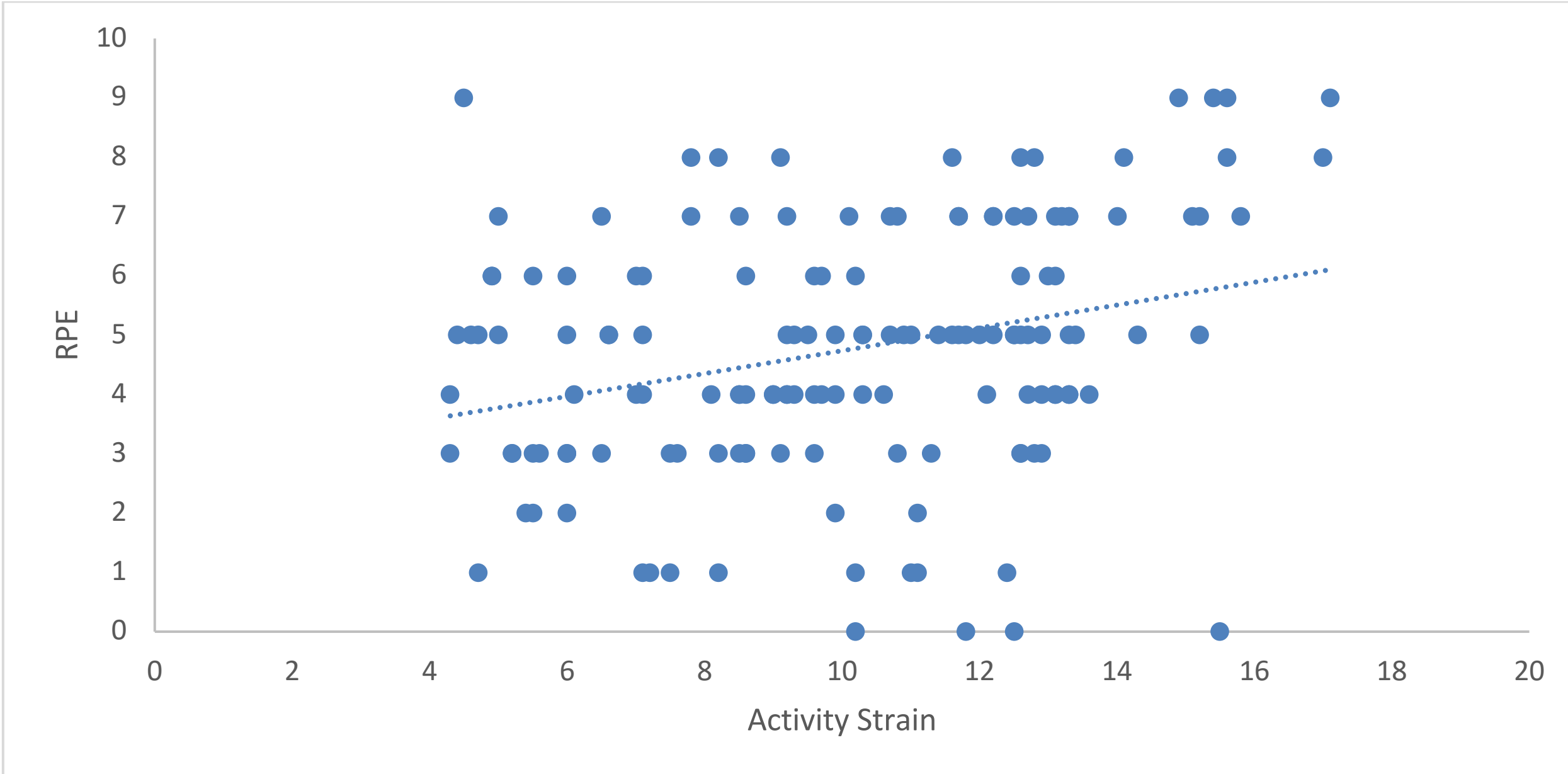

Supplementary Figure 2. Scatter plot of all observations across rating of perceived exertion (RPE) and strain variables.
